# TiO_2_ Nanotubes Decorated with Mo_2_C for Enhanced Photoelectrochemical Water-Splitting Properties

**DOI:** 10.3390/ma16186261

**Published:** 2023-09-18

**Authors:** Siti Nurul Falaein Moridon, Khuzaimah Arifin, Mohamad Azuwa Mohamed, Lorna Jeffery Minggu, Rozan Mohamad Yunus, Mohammad B. Kassim

**Affiliations:** 1 Fuel Cell Institute, Universiti Kebangsaan Malaysia, Bangi 43600, Selangor, Malaysia; 2 Department of Chemical Sciences, Faculty of Science and Technology, Universiti Kebangsaan Malaysia, Bangi 43600, Selangor, Malaysia

**Keywords:** titanium dioxide, anodization, self-doping, cocatalyst, Mo_2_C

## Abstract

The presence of Ti^3+^ in the structure of TiO_2_ nanotube arrays (NTs) has been shown to enhance the photoelectrochemical (PEC) water-splitting performance of these NTs, leading to improved results compared to pristine anatase TiO_2_ NTs. To further improve the properties related to PEC performance, we successfully produced TiO_2_ NTs using a two-step electrochemical anodization technique, followed by annealing at a temperature of 450 °C. Subsequently, Mo_2_C was decorated onto the NTs by dip coating them with precursors at varying concentrations and times. The presence of anatase TiO_2_ and Ti_3_O_5_ phases within the TiO_2_ NTs was confirmed through X-ray diffraction (XRD) analysis. The TiO_2_ NTs that were decorated with Mo_2_C demonstrated a photocurrent density of approximately 1.4 mA cm^−2^, a value that is approximately five times greater than the photocurrent density exhibited by the bare TiO_2_ NTs, which was approximately 0.21 mA cm^−2^. The observed increase in photocurrent density can be ascribed to the incorporation of Mo_2_C as a cocatalyst, which significantly enhances the photocatalytic characteristics of the TiO_2_ NTs. The successful deposition of Mo_2_C onto the TiO_2_ NTs was further corroborated by the characterization techniques utilized. The utilization of field emission scanning electron microscopy (FESEM) allowed for the observation of Mo_2_C particles on the surface of TiO_2_ NTs. To validate the composition and optical characteristics of the decorated NTs, X-ray photoelectron spectroscopy (XPS) and UV absorbance analysis were performed. This study introduces a potentially effective method for developing efficient photoelectrodes based on TiO_2_ for environmentally sustainable hydrogen production through the use of photoelectrochemical water-splitting devices. The utilization of Mo_2_C as a cocatalyst on TiO_2_ NTs presents opportunities for the advancement of effective and environmentally friendly photoelectrochemical (PEC) systems.

## 1. Introduction

The extreme reliance on fossil fuels for energy generation since the industrial revolution has triggered a global energy crisis and various other environmental problems [1,2]. Therefore, reducing energy dependence on fossil fuels through the provision of clean and renewable energy sources is urgent. One alternative is to use clean and green hydrogen (H_2_) directly produced from water molecules using solar light energy, known as the photoelectrochemical (PEC) process. H_2_ can be used as fuel in an electrochemical fuel cell device to produce electricity, with pure water as the only byproduct.

In the PEC process, semiconductor materials are employed as photoelectrodes [2]. To date, numerous semiconductor materials, such as TiO_2_ [3,4,5], Co_3_O_4_ [6,7], WO_3_ [8], Cu_2_O [9], and Fe_2_O_3_ [10], have been investigated as photoelectrode materials. Among them, TiO_2_ has garnered considerable attention due to its photoactivity, low cost, excellent chemical stability, and abundance in nature [11]. However, TiO_2_ can only be stimulated by UV light, which accounts for only 3–5% of solar energy radiation, because of its large band gap, further causing quick recombination of photoinduced electron-hole pairs as well as inefficient charge carrier separation [11]. Therefore, various methods have been used to improve the PEC performance and photocatalytic activity of TiO_2_, including morphology modification [12,13,14], synthesis of composite heterojunctions with other materials [15], ion doping [16,17], facet engineering [18], and cocatalyst addition [19].

In terms of morphology, TiO_2_ nanotube arrays (NTs) have attracted attention due to their high specific surface area, excellent adsorption capacity, and good structural properties for electron transport. Several methods have been employed to fabricate TiO_2_ NT photoelectrodes, and electrochemical anodization is considered one of the most promising methods for fabricating a highly ordered NT structure [20]. Although the electrochemical anodization method is considered a promising fabrication method for producing ordered TiO_2_ NTs with defect engineering and doping, the PEC performance of fabricated TiO_2_ NTs still does not reach satisfactory levels due to their restricted light harvesting and the high resistance at the interface between the nanotubes and the substrate. Therefore, a synergistic approach combining various strategies, such as the formation of heterojunctions with other semiconductor materials or the addition of cocatalyst materials, could prove excellent for obtaining TiO_2_ NTs with efficient light harvesting and charge separation for high PEC water-splitting performance. Currently, 2D MXenes have been investigated as promising catalysts or cocatalysts in many applications. The nomenclature “MXene” has been used to represent a group of compounds that includes transition metal carbides, nitrides, and carbonitrides. The nomenclature “MXene” is derived from its chemical composition, wherein the symbol “M” represents a transition metal, “X” signifies carbon and/or nitrogen, and the suffix “ene” refers to its two-dimensional structural arrangement [21]. Among the reported MXene materials, dimolybdenum carbide (Mo_2_C) has been reported to show excellent electrocatalytic performance in the hydrogen evolution reaction (HER) [21]. It has an electronic density of states comparable to that of Pt and excellent electrical conductivity. In addition to being used as a catalyst for the HER, Mo_2_C has also been investigated for photocatalytic water splitting for hydrogen generation [22]. Shen et al. reported that the Mo_2_C/CdS nanocomposite produced a photocurrent density 7.83 times higher than that of pure CdS [23]. Furthermore, Yue et al. reported that dandelion-like TiO_2_ nanoparticles with 1% Mo_2_C were able to produce H_2_ with a production rate of 39.4 mmol h^−1^ g^−1^, which is 25 times that obtained with pristine TiO_2_ [22].

Although Mo_2_C has been shown to be a good cocatalyst for TiO_2_, the photocatalytic performance of TiO_2_ NTs on Ti foil substrates with Mo_2_C has yet to be reported. This study presents the effectiveness of TiO_2_ NTs with Mo_2_C incorporated as a cocatalyst for PEC water-splitting applications. Here, TiO_2_ NTs were prepared by two-step electrochemical anodization, and Mo_2_C was inserted by dip coating the NTs into Mo_2_C precursors of various concentrations for various dipping times. Our findings indicated that the combined effect of multiple PEC improvement strategies could offer a versatile and systematic way to overcome the intrinsic and extrinsic limitations of TiO_2_ NTs for PEC water-splitting applications.

## 2. Materials and Methods

### 2.1. Materials

Titanium foil (0.127 nm thickness, obtained from Sigma Aldrich, St. Louis, MI, USA), molybdenum carbide (Mo_2_C) (99%, obtained from Sigma Aldrich), Pt mesh (99%, obtained from Sigma Aldrich), ethylene glycol (analytical grade, obtained from Merck, Darmstadt, Germany), ammonium fluoride NH_4_F (analytical grade, obtained from Merck, Germany), distilled water, ethanol (analytical grade, obtained from QReC, Kuala Lumpur, Malaysia) and sodium sulfate (Na_2_SO_4_) were used. All chemicals were used as received from the manufacturer without additional purification.

### 2.2. Fabrication of TiO_2_ NTs and TiO_2_ NTs Decorated with Mo_2_C

The TiO_2_ NTs were fabricated using a multiple anodization technique [24]. First, cleaned Ti foil (1.5 cm × 1.5 cm) was used as the anode, and Pt foil was used as the counter electrode connected to a power supply at a voltage of 50 V for one hour. Ethylene glycol containing 0.3 vol. % NH_4_F and 2 vol. % distilled water was used as the electrolyte. The anodized film was then sonicated in a mixture of ethanol and distilled water (1:1) for 5 min to clean dirt away from the openings of the grown nanotubes. Subsequently, the Ti foil underwent a second anodization process for 30 min in the same electrolyte at the same voltage. Then, ethanol and distilled water were used to flush the samples. The anodized samples were then annealed at 450 °C for 3 h at a ramping rate of 2 °C/min. The best TiO_2_ NTs that achieved the highest photocurrent were then dip coated in an ethanol/distilled water mixture containing highly dispersed Mo_2_C at four different concentrations of 5 g/L, 10 g/L, 15 g/L and 20 g/L, and the obtained samples were labeled S-1, S-2, S-3 and S-4, respectively.

### 2.3. Characterization

X-ray diffraction (XRD) patterns were acquired via a Bruker D-8 Advance (Ettlingen, Germany, Equipment sourced by Bruker Malaysia), and X-ray photoelectron spectroscopy (XPS) was performed using a Kratos/Shimadzu instrument (model: Axis Ultra DLD) (Milton Keynes, UK, Equipment sourced by Shimadzu Malaysia) to determine the chemical phases present in the crystalline substances. The XRD patterns were analyzed using X’Pert HighScore software (Version 2.2b). To investigate the topographic nature of the surface, field emission scanning electron microscopy (FESEM) was carried out using a Zeiss Merlin Compact microscope (Oberkochen, Germany, Equipment sourced by Zeiss Malaysia). The optical properties were analyzed using a Perkin Elmer ultraviolet/visible/near-infrared spectrophotometer (UV–VIS-NIR) (model: Lambda 950) (Waltham, MA, USA, Equipment sourced by Perkin Elmer Malaysia).

### 2.4. PEC Property Measurements

An Ametek Versastat 4 was used to carry out the PEC analysis. An exposed area of 1 cm^2^ was employed for testing the thin films that served as the working electrode in a PEC cell. The counter electrode consisted of a platinum wire; the reference electrode was a Ag/AgCl electrode. The counter electrode measured the potential difference between the two electrodes. In these experiments, 0.5 M Na_2_SO_4_ (pH 6.7) was used as the electrolyte. The current density on the thin film surfaces was measured in the dark and under solar AM 1.5 illumination using a xenon lamp (Oriel with an intensity of 100 mW cm^−2^). Linear sweep voltammetry (LSV) was conducted from 0 to +1.0 V versus Ag/AgCl in 0.5 M Na_2_SO_4_ at a scan rate of 5 mV s^−1^. To obtain a deeper understanding of the charge transport behavior shown by the synthesized photoanodes, Mott-Schottky analysis was performed at 1 kHz. This allowed for calculation of the charge carrier densities, as well as the conduction band (CB). The electrochemical impedance spectra (EIS) Nyquist plots were constructed by utilizing 10 mV sinusoidal perturbations at a frequency of 100 kHz.

## 3. Results and Discussion

### 3.1. Physical Characterization of TiO_2_ NTs

**Figure 1:**
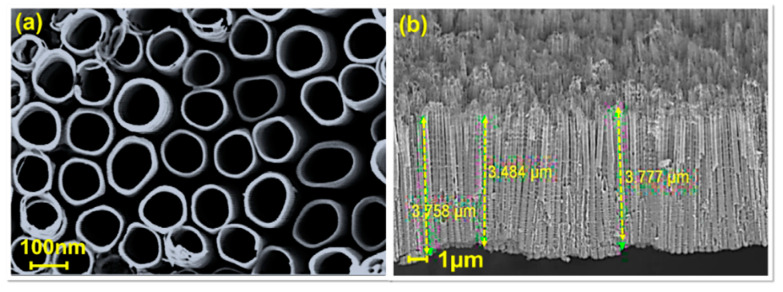
FESEM images of the (**a**) surface morphology and (**b**) cross-section of TiO_2_ NTs.

To thoroughly investigate the growth of TiO_2_ NTs, a detailed analysis was conducted comprising analysis of the morphology, crystal phase, crystallinity, and optical properties. Figure 1 shows the FESEM results that capture the microstructure of the TiO_2_ NT sample.

Based on the FESEM images, the TiO_2_ NT sample clearly exhibited non-interconnected single tubes (Figure 1a). The diameter of the TiO_2_ NTs was ~151–160 nm. Figure 1b displays cross-sectional views of TiO_2_ NTs. The length of the TiO_2_ NTs was ~3.4–3.8 µm.

**Figure 2:**
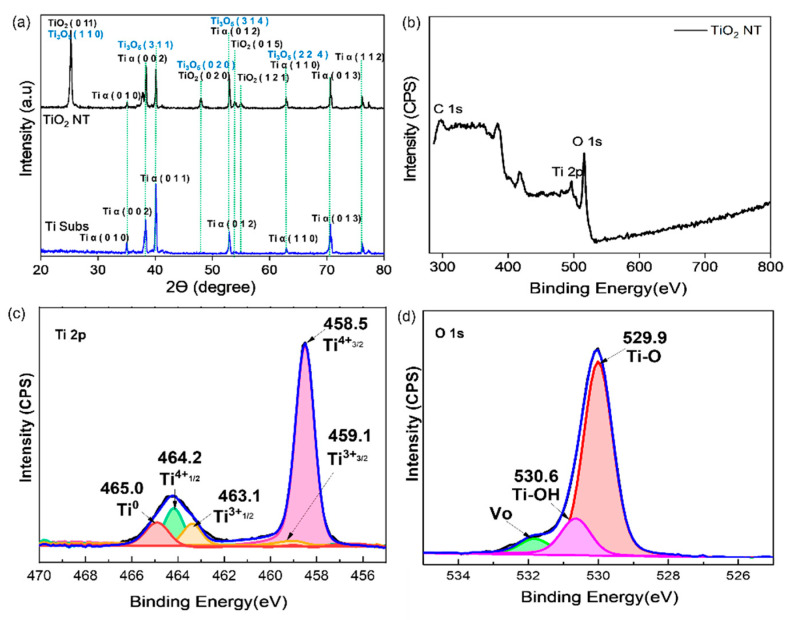
(**a**) XRD patterns and (**b**) XPS survey spectrum of TiO_2_ NTs; (**c**) Ti 2p and (**d**) O 1s spectra of TiO_2_ NTs.

Next, XRD analysis was carried out to identify the phases and determine the chemical composition. Figure 2a shows the XRD patterns of the Ti foil substrate and TiO_2_ NT sample.

The spectra show Ti alpha diffraction peaks representing the Ti foil at 35.01°, 38.27°, 40.10°, 52.99°, 62.89°, 70.66° and 76.18°, corresponding to the (0 1 0), (0 0 2), (0 1 1), (0 1 2), (0 1 3), (1 1 0) and (1 1 2) planes, respectively. However, when the sample was anodized, anatase peaks appeared at 25.28°, 47.83°, 53.09° and 55.02°, corresponding to the (0 1 1), (0 2 0), (0 1 5) and (1 2 1) planes, respectively. The intensity of the Ti alpha peaks was reduced because the surface of the titanium substrate was oxidized during the anodization process, which resulted in the formation of a layer of anatase titanium oxide. The XRD patterns found in this study are similar to those found in Quiroz et al., (2015), which contain a tri-titanium pentoxide (Ti_3_O_5_) phase [25]. Based on the XRD library patterns, the Ti alpha peaks overlapped with the Ti_3_O_5_ peaks at 38.27°, 52.99° and 70.66° and overlapped with some anatase peaks at 25.28° and 47.83°.

Figure 2b shows the results of the XPS analysis conducted to verify the presence of Ti_3_O_5_ in the TiO_2_ NT samples. The peaks at ~464 eV and 458 eV correspond to Ti 2p, and the Ti 2p peaks of Ti^3+^ were observed at binding energies of 463.1 eV (Ti^3+^ 2p^3/2^) and 459.1 eV (Ti^4+^ 2p^1/2^). The Ti 2p peaks of Ti^4+^ appeared at 458.5 eV (Ti^4+^ 2p^3/2^) and 464.2 eV (Ti^4+^ 2p^1/2^). The XPS spectra of TiO_2_ NTs revealed a modest shift in position and a change in the size of the original peaks of TiO_2_ NTs from those in a previous study after self-doping with Ti^3+^. The observed peak shift indicates that the self-doping of Ti with Ti^3+^ affected its electronic state. As a consequence of this process, some of the Ti^4+^ ions in the lattice are believed to have been replaced by Ti^3+^ ions. Furthermore, the decrease in the Ti^4+^ peaks suggests that there was less TiO_2_ present in the sample. The creation of oxygen vacancies in the surface layer during the multistep anodization procedure can be deduced to be responsible for the diminishing area of the Ti^4+^ species peak [26]. Furthermore, the peaks at binding energies of 529.9 eV and 530.6 eV were ascribed to lattice oxygen for TiO_2_ NTs. The O 1s spectra provided more evidence demonstrating that more oxygen defects were present in the TiO_2_ NTs [27]. The percentage of atomic oxygen vacancies for TiO_2_ NTs was 6.25%. The XPS results agreed with the XRD results, suggesting that more Ti^3+^ was produced during the anodization process.

**Figure 3:**
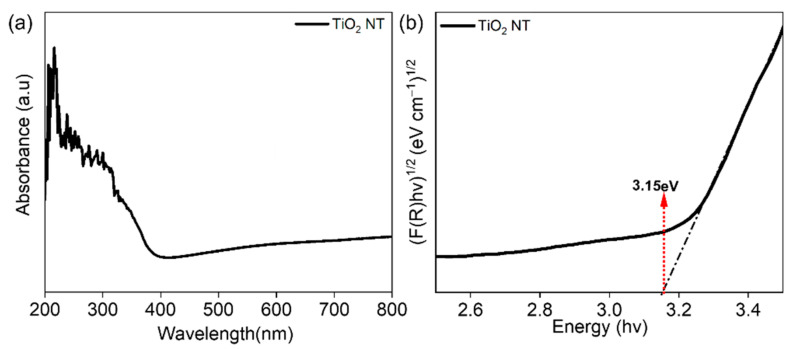
(**a**) Absorption spectra and (**b**) band gap determination by Kubelka–Munk plot analysis.

Figure 3a shows the UV–Vis absorption spectra over the wavelength range from 250 to 800 nm, showing that the TiO_2_ NTs had higher absorption in the UV range. Next, the band gap of the sample was calculated using Kubelka–Munk theory, and the value for the TiO_2_ NTs was 3.15 eV [3].

### 3.2. Physical Characterization of Mo_2_C as a Cocatalyst Decorated on TiO_2_ NTs

Mo_2_C was added to TiO_2_ NTs using a dip coating technique. Dip coating is a simple, dependable, and robust process that can be used to cover almost any substrate material by immersing it in a solution and then removing it to drip dry to form a conformal coating.

**Figure 4:**
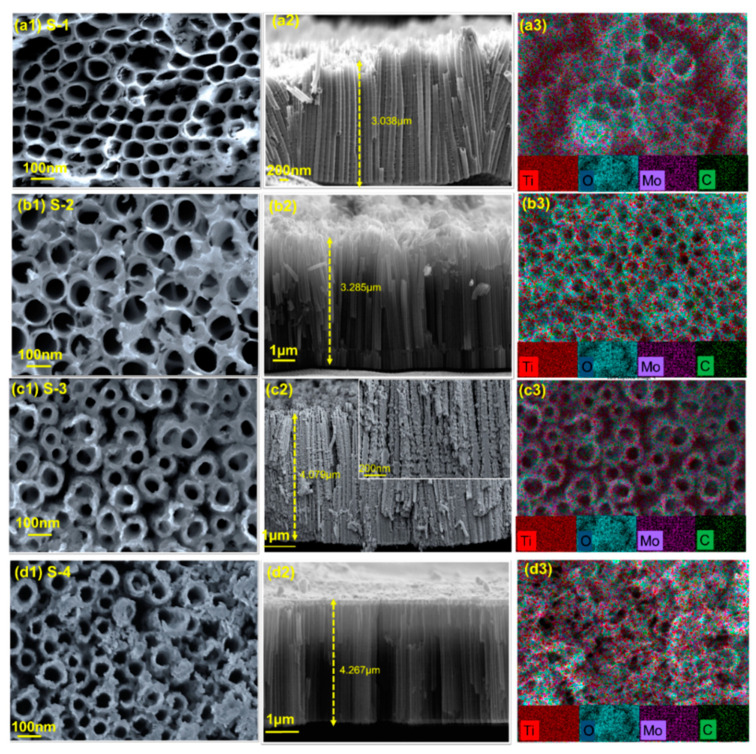
FESEM images of the surface and cross-section, as well as EDX mapping of samples at different concentrations (**a1**–**a3**) S-1, (**b1**–**b3**) S-2, (**c1**–**c3**) S-3 and (**d1**–**d3**) S-4.

FESEM analysis was performed to study the effect of different concentrations of the Mo_2_C precursor on the morphology of TiO_2_ NTs. Figure 4(a1–d3) shows micrographs of TiO_2_ NTs for different precursor Mo_2_C concentrations.

As shown in Figure 4, increasing the concentration resulted in an increasing deposition amount. Figure 4(a1,b1) illustrate that the distribution of Mo_2_C on the surface of the TiO_2_ NTs was not uniform. Figure 4(c1) shows that Mo_2_C was well distributed inside the TiO_2_ NT tubes at a concentration of 15 g/L, which was crucial for the photoelectrode activity. Increasing the concentration to 20 g/L resulted in large nanoclusters of Mo_2_C blocking most TiO_2_ NTs (Figure 4(d1)). Next, Figure 4(a2–d2) illustrates cross-sectional views of the decorated Mo_2_C on the TiO_2_ NTs. The length of the TiO_2_ NTs increased as the concentration of Mo_2_C increased; this finding may support the idea that Mo_2_C is distributed on the upper openings of the TiO_2_ NTs. In addition, an image of the cross-section of sample S-3 can be seen in the inset of Figure 4(c2); this image demonstrates that Mo_2_C decorated the outside wall of the tubes. Energy-dispersive X-ray spectroscopy (EDX) mapping and cross-section analysis were performed to determine the distribution of Mo_2_C in sample S-3 with a concentration of 15 g/L. The findings are shown in Figure 4(a3–d3), suggesting that Mo_2_C was equally dispersed over the TiO_2_ NT surface and in the interstices. This indicates that Mo_2_C was efficiently distributed across the sample, resulting in a uniform distribution.

**Figure 5:**
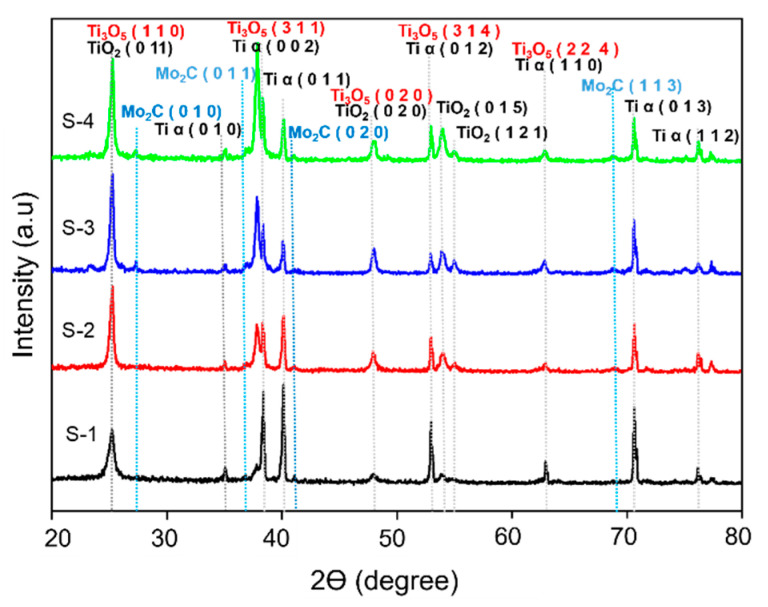
XRD patterns of S-1, S-2, S-3 and S-4.

The XRD patterns of TiO_2_ NTs after deposition of Mo_2_C are presented in Figure 5.

The diffraction peaks at 25.1°, 37.8°, 48.0°, 52.9° and 62.3° in the pattern of bare TiO_2_ NTs were identified as corresponding to the planes of anatase TiO_2_ and Ti_3_O_5_ phases (JCPDS nos. 98-009-4632 and 98-007-1775). Upon deposition of Mo_2_C nanoparticles, the patterns displayed additional peaks at 27.2°, 37.2°, 38.3°, 41.1° and 68.8°, which correspond to the standard diffraction peaks of Mo_2_C (JCPDS no. 98-006-1705). These findings are consistent with the FESEM results, in which increasing the concentration of Mo_2_C leads to increases in the amount of deposited Mo_2_C and the intensity of the peaks. The observed peaks indicate the successful deposition of Mo_2_C nanoparticles onto the TiO_2_ NT surface, which can potentially enhance the PEC properties of the material.

**Figure 6:**
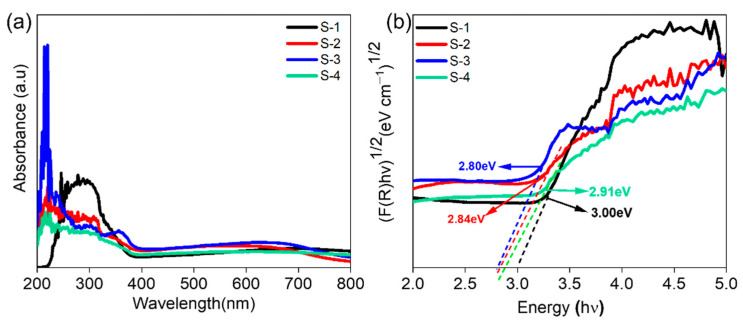
(**a**) UV–Vis absorption spectra and (**b**) Kubelka–Munk plots for band gap determination of S-1, S-2, S-3 and S-4.

The UV–Vis absorption spectra of Mo_2_C/TiO_2_ NTs with various concentrations are presented in Figure 6a. The TiO_2_ NTs loaded with Mo_2_C nanoparticles exhibited a broader absorption in the visible light region (450 nm to 800 nm) compared to pure TiO_2_ NTs. Among the samples, S-3 showed the highest absorption and thus had the highest PEC activity. The band gap of the samples is displayed in Figure 6b, revealing that the Mo_2_C-loaded TiO_2_ NTs had a lower band gap than the pure TiO_2_ NTs.

The band gap of S-3 was determined to be ~2.80 eV, the smallest among the samples. Previous reports suggest that higher absorption in the visible region corresponds to better PEC water-splitting activity. Fine-tuning the band gap and band locations is necessary when creating visible light-responsive photocatalysts for hydrogen production.

### 3.3. PEC Properties of TiO_2_ NTs and Mo_2_C as a Cocatalyst Decorated on TiO_2_ NTs

Mo_2_C has garnered interest in the field of PEC applications due to its exceptional stability in challenging environments and remarkable electrical conductivity, making it a promising cocatalyst for such purposes. Mo_2_C applied onto TiO_2_ NTs has been observed to function as an electron transfer mediator, thereby facilitating the separation of photogenerated charge carriers and resulting in an improved overall PEC performance of TiO_2_ NTs. The hybridization of TiO_2_ NTs with Mo_2_C has been found to exhibit a synergistic effect, whereby the distinctive characteristics of each material are combined to overcome the constraints of TiO_2_ NTs. This discussion explores the PEC characteristics of TiO_2_ NTs and TiO_2_ NTs that have been decorated with Mo_2_C as a cocatalyst.

**Figure 7:**
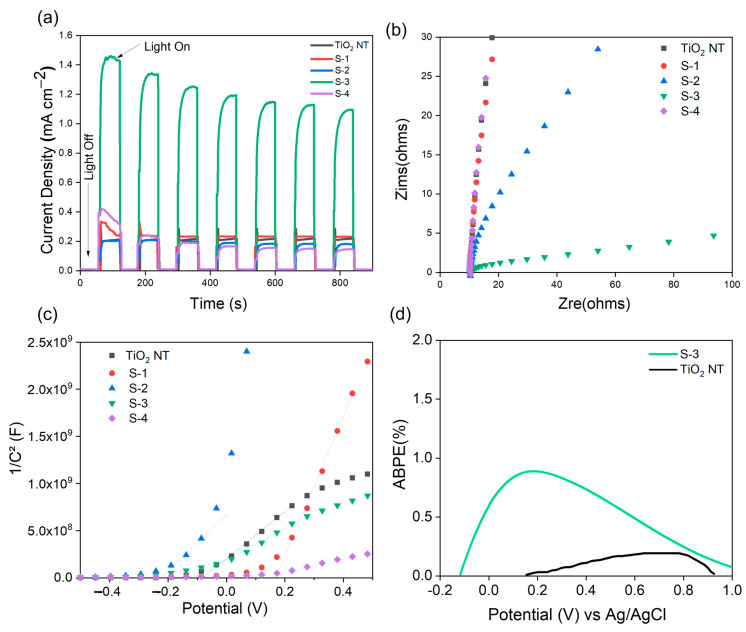
(**a**) Photocurrent density and stability under light chopping of TiO_2_ NTs and S1–S4; (**b**) EIS spectra of the TiO_2_ NT and S1–S4 samples; (**c**) N-type Mott–Schottky plots of TiO_2_ NTs and S1–S4; (**d**) ABPE % of TiO_2_ NTs and S-3.

The correlation between the TiO_2_ NTs with Mo_2_C as a cocatalyst and the PEC behavior of TiO_2_ NTs was investigated by chronoamperometric measurements under light chopping, and the test was carried out in 0.5 M Na_2_SO_4_ at a bias potential of 0.7 V vs. Ag/AgCl in the presence and absence of illumination (light-off and light-on). The concentration of Mo_2_C varied, with values of 5 g/L (S-1), 10 g/L (S-2), 15 g/L (S-3) and 20 g/L (S-4), as shown in Figure 7a.

All samples demonstrated a satisfactory photocurrent density as well as a good level of stability after 900 s. The photocurrent density of the TiO_2_ NT sample was determined to be 0.21 mA cm^−2^, and this value of photocurrent increased approximately one-fold when compared to the value of pure TiO_2_ NT due to the presence of oxygen vacancy defects, as reported in previous work [28,29,30]. Meanwhile, the photocurrent densities produced by samples S-1, S-2 and S-4 were similar to that of bare TiO_2_ NTs. The significant photocurrent density produced by sample S-3 had a value of ~1.4 mA cm^−2^, nearly five times higher than that of bare TiO_2_ NTs.

EIS is a trustworthy method for investigating the charge transfer and recombination rate at semiconductor electrolyte interfaces, where “Zre” is the real portion and “Zim” is the imaginary part. Due to the relationship between the arc of the circle and the charge transfer resistance, the Nyquist plots provide sufficient information on the charge transfer. In the Nyquist plot, a smaller arc indicates greater charge carrier separation and higher charge transfer efficiency (conductivity) [30]. Figure 7b shows that sample S-3 has the smallest semicircle radius. This indicates that photogenerated electron-hole pairs are more effectively separated and that electrons may more easily cross the valence band in response to a relatively low-energy excitation. As a consequence, the charge transfer in S-3 is enhanced, proving the presence of a large separation between the holes and electrons.

The Mott–Schottky (M–S) curves of all photoelectrode samples are shown in Figure 7c. The flat band potential (*E*_fb_) was estimated by projecting the linear section of the plots onto the potential axis. In addition, the donor density (*N*_D_) was calculated using the slope of the M–S curves and Equation (1) obtained from previous work [25,30]. The *N*_D_ and *E*_fb_ values determined are reported in Table 1, and the *E*_fb_ values of sample S-3 are less negative, which indicates an upward shift of the Fermi level [30,31].

(1) $$N_{D}=\left(\frac{2}{e\varepsilon \varepsilon_{o}A^{2}}\right){\left[\frac{d\left(\frac{1}{C2}\right)}{dE}\right]}^{-1}$$

where $\left[\frac{d\left(\frac{1}{C2}\right)}{dE}\right]$ is the slope of the tangent line in the Mott–Schottky plot, *e* is the electron charge, *ε* is the dielectric constant of the TiO_2_ film, *ε*^0^ is the vacuum permittivity, and *A* is the surface area of the TiO_2_ NT thin film electrode.

The efficiency with which a PEC cell converts light into electricity is quantified by the applied bias photon-to-current efficiency (ABPE). To assess how well a PEC cell converts solar energy into a usable form, the ABPE test is crucial. This method is useful for comparing the efficiency of various materials in converting light into electricity and for determining the efficacy of individual materials. To further improve the PEC cell design, the ABPE may be utilized to investigate how an applied bias affects the cell output. In conclusion, the ABPE is a useful metric for assessing PEC cell performance, yielding crucial data for improving future solar energy conversion technologies. Figure 7d shows the ABPE measurements of TiO_2_ NTs and S-3. The ABPE value was calculated using Equation (2) [32,33]:

ABPE % = [*J*_p_ (*E*_0_ rev − *E*_app_)/*I*_0_] × (100) (2)

where *J*_p_ is the photocurrent density (mA/cm^2^), *I*_0_ is the illumination intensity (mW/cm^2^), *E*_0_ rev is the standard reversible potential for water splitting (1.23 V), and *E*_app_ is the applied potential. The highest ABPE value for TiO_2_ NTs is 0.19% at 0.8 V, while that of S-3 is 0.89% at 0.2 V. Next, the solar-to-hydrogen efficiency (STH) was also calculated for PEC water splitting with a visible light source of irradiance 100 mW cm^−2^ using Equation (3) [32,33]:

(3) $$STH\left(\%\right)=J_{P}\frac{1.23-V_{App}}{P}\;\times \;100$$

where *J*_p_ is the photocurrent density (mA/cm^2^), *V*_app_ is the applied potential, and *P* is the intensity of the light source. The maximum STH % for TiO_2_ NTs is 0.05%, while that for Mo_2_C/TiO_2_ NTs (S-3) is 0.32%, as shown in Table 2.

**Figure 8:**
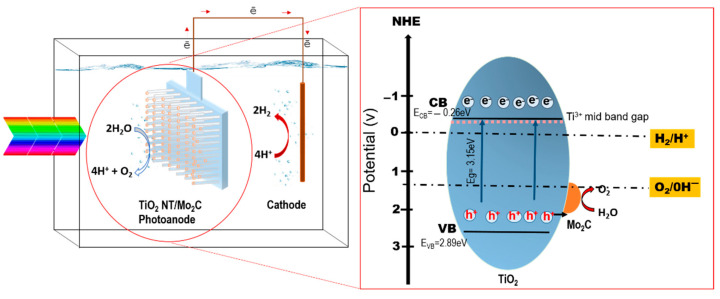
Proposed mechanism of Mo_2_C as a cocatalyst in TiO_2_ NTs self-doped by Ti^3+^.

The increased PEC properties of TiO_2_ NTs decorated with Mo_2_C are further explained by the proposed mechanism shown in Figure 8.

In this work, oxygen vacancies produce localized electronic states inside the energy gap, which correspond to Ti^3+^ species, which are in the mid-band gap. These restricted states may serve as traps or recombination sites for electrons and holes created by photons. The Mo_2_C deposited onto the TiO_2_ photoanode may serve as a cocatalyst to accelerate the HER and oxygen evolution reaction (OER), leading to a higher efficiency in PEC water splitting. Moreover, the Mo_2_C cocatalyst may minimize the energy barrier for charge transfer and avoid charge recombination, resulting in an increased photocurrent and better stability.

Upon irradiation with solar light, the TiO_2_ electrons may be excited into the CB during the process, and electrons move from the TiO_2_ photoanode to the cathode, resulting in the reduction of protons to hydrogen at the cathode. Mo_2_C works as a cocatalyst, boosting the oxidation of water molecules by facilitating the flow of holes from the TiO_2_ photoanode surface to the water molecules. The Mo_2_C catalyst helps reduce the energy barrier for the OER, which leads to a decrease in the overpotential needed to drive the reaction. This may lead to an increase in the rate of the reaction and consequently a greater efficiency of the entire water-splitting process.

The method for achieving very uniform Mo_2_C nanoparticles distributed on both the inside and outside vertically aligned TiO_2_ NTs via the dip coating deposition process has great promise. These novel interactions of Mo_2_C/TiO_2_ NTs dramatically enhance both the light absorption and the PEC activity under visible light illumination by 5-fold compared to those of pure TiO_2_ NTs. These characterization results are consistent with an improved Mo_2_C/TiO_2_ NT performance, although the result is not as incredible as that reported by previous researchers on the photocatalytic effect of Mo_2_C for pristine powder TiO_2_ [34]. There is still much room to improve the performance, such as by optimizing the length and tube diameter of TiO_2_ NTs so that they are suitable for Mo_2_C diffusion, as well as the Mo_2_C deposition methods.

## 4. Conclusions

The implementation of Mo_2_C as a cocatalyst led to a significant enhancement in the photocurrent density of TiO_2_ NTs. The photocurrent density of the modified TiO_2_ NTs was observed to be significantly enhanced by a factor of five when compared to the unmodified TiO_2_ NTs. The implications of these findings are of great importance for the further development of environmentally sustainable PEC water-splitting technologies, specifically in the domain of hydrogen production. The successful decoration of TiO_2_ NTs with Mo_2_C was confirmed through XRD and FESEM analysis. Moreover, the incorporation of Mo_2_C has been observed to significantly decrease the band gap and enhance the light absorption capabilities of TiO_2_ NTs. Significantly, it was determined that the most favorable concentration of Mo_2_C was 15 g/L (S-3), exhibiting the highest photoelectrochemical efficiency. In general, this study provides insights into the possible utilization of Mo_2_C-decorated TiO_2_ NTs, specifically the Ti_3_O_5_ phase, for enhancing the effectiveness and efficacy of photoelectrochemical systems. The utilization of Mo_2_C as a cocatalyst in PEC water-splitting applications is highly promising due to several factors. These include the achievement of enhanced photocurrent density, the confirmation of Mo_2_C’s presence through X-ray diffraction (XRD) analysis, the reduction of the band gap, and the determination of an optimal concentration.

## Figures and Tables

**Table 1:** Flat band potential (*E*_fb_) and donor density (*N*_D_) of TiO_2_ NTs and S-3.

Sample	*N*_D_ (×10^9^ cm^−3^)	*E*_fb_ (V)	Band Gap (eV)
TiO_2_ NT	3.7918	−0.08	3.17
S-3	3.2629	−0.12	2.80

**Table 2:** Measured parameters of the PEC cell.

Sample Details	Photocurrent Density (mA cm^−2^) at 1 V vs. Ag/AgCl	Solar to Hydrogen Conversion Efficiency, (η %)
TiO_2_ NT	0.23	0.05
S-3	1.4	0.32

## Data Availability

The authors do not have permission to share data.
